# The Kinetic Mechanism of 3′-5′ Exonucleolytic Activity of AP Endonuclease Nfo from *E. coli*

**DOI:** 10.3390/cells11192998

**Published:** 2022-09-26

**Authors:** Svetlana I. Senchurova, Aleksandra A. Kuznetsova, Alexander A. Ishchenko, Murat Saparbaev, Olga S. Fedorova, Nikita A. Kuznetsov

**Affiliations:** 1 Institute of Chemical Biology and Fundamental Medicine, Siberian Branch of Russian Academy of Sciences, 630090 Novosibirsk, Russia; 2 Group «Mechanisms of DNA Repair and Carcinogenesis», Equipe Labellisée LIGUE 2016, CNRS UMR9019, Université Paris-Saclay, Gustave Roussy Cancer Campus, CEDEX, F-94805 Villejuif, France; 3 Department of Natural Sciences, Novosibirsk State University, 630090 Novosibirsk, Russia

**Keywords:** DNA repair, apurinic/apyrimidinic endonuclease, pre-steady-state kinetics, fluorescence

## Abstract

*Escherichia coli* apurinic/apyrimidinic (AP) endonuclease Nfo is one of the key participants in DNA repair. The principal biological role of this enzyme is the recognition and hydrolysis of AP sites, which arise in DNA either as a result of the spontaneous hydrolysis of an N-glycosidic bond with intact nitrogenous bases or under the action of DNA glycosylases, which eliminate various damaged bases during base excision repair. Nfo also removes 3′-terminal blocking groups resulting from AP lyase activity of DNA glycosylases. Additionally, Nfo can hydrolyze the phosphodiester linkage on the 5′ side of some damaged nucleotides on the nucleotide incision repair pathway. The function of 3′-5′-exonuclease activity of Nfo remains unclear and probably consists of participation (together with the nucleotide incision repair activity) in the repair of cluster lesions. In this work, using polyacrylamide gel electrophoresis and the stopped-flow method, we analyzed the kinetics of the interaction of Nfo with various model DNA substrates containing a 5′ single-stranded region. These data helped to describe the mechanism of nucleotide cleavage and to determine the rates of the corresponding stages. It was revealed that the rate-limiting stage of the enzymatic process is a dissociation of the reaction product from the enzyme active site. The stability of the terminal pair of nucleotides in the substrate did not affect the enzymatic-reaction rate. Finally, it was found that 2′-deoxynucleoside monophosphates can effectively inhibit the 3′-5′-exonuclease activity of Nfo.

## 1. Introduction

The formation of apurinic/apyrimidinic (AP) sites in DNA is a relatively frequent event and can occur as many as 10,000 times per day [1,2,3]. AP sites have a cytotoxic effect because they inhibit replicative DNA polymerases and also lead to DNA mutations. The repair of DNA-containing AP sites in vivo is performed via the base excision repair pathway [4,5,6]. Over the course of base excision repair, hydrolytic cleavage of the phosphodiester bond takes place on the 5′ side of an AP site; this process is catalyzed by AP endonucleases.

In *Escherichia coli*, two AP endonucleases have been found, which differ in their amino acid sequence and structural organization: exonuclease III (Xth) and endonuclease IV (Nfo). Nfo is a multifunctional EDTA-resistant enzyme possessing AP endonuclease, 3′-phosphodiesterase, and 3′-5′-exonuclease activities [7,8]. This 30 kDa protein carries 3 Zn^2+^ ions in its active site; these are necessary for its catalytic activity [9]. The *nfo* gene is under the control of the soxRS system and is induced in the presence of radicals O_2_^•−^ and NO^•^ [10]. Deletion of the *nfo* gene in *E. coli* cells enhances its sensitivity to antitumor drug bleomycin and to an organic peroxide called *tert*-butyl hydroperoxide (t-BuO_2_H) [11]. However, *E. coli*, harboring a knockout of genes of DNA glycosylases specific to oxidized bases, is insensitive to oxidizing agents and ionizing radiation [12,13,14], pointing to the existence of an alternative repair pathway for these lesions. Indeed, such an alternative repair pathway, i.e., nucleotide incision repair (NIR), has been found in a number of organisms, ranging from bacteria to mammals [15]. In *E. coli* cells, NIR is initiated by the Nfo enzyme [16]. Furthermore, just as most AP endonucleases, Nfo has 3′-5′-exonuclease activity [17]. A cluster lesion repair scheme has been proposed, where the NIR activity and 3′-5′-exonuclease activity of Nfo are necessary to the effective removal of lesions located in both DNA strands; this mechanism prevents the formation of double-strand breaks, which are the most destructive form of DNA damage in the cell [18].

Thus, it can be concluded that Nfo plays a major role in the repair of AP sites, 3′ blocking ends, and certain types of oxidative damage, and this variety of substrates points to the multifunctionality of this enzyme. This raises the question of how this AP endonuclease, which possesses a single active site, controls its own substrate specificity. The AP site (its main substrate) and the damaged nucleotides substantially differ in structure and, moreover, during the 3′-5′-exonuclease activity, the enzyme recognizes and hydrolyzes phosphodiester bonds in intact nucleic acids consisting only of natural nucleotides.

**Figure 1:**
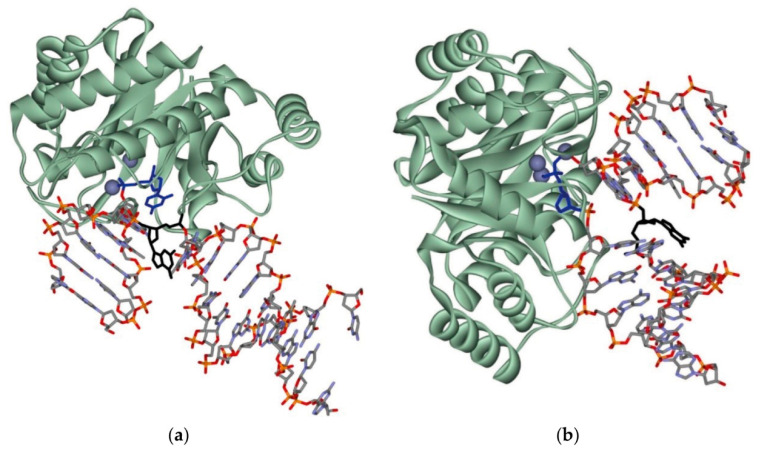
The crystal structure of the exonuclease complex of Nfo with an NIR-mediated DNA (Protein Data Bank [PDB] ID 4K1G) (**a**) and the structure of a complex of Nfo with a DNA substrate containing an F-site (PDB ID 1QUM) (**b**).

According to X-ray diffraction data, if we compare (i) interaction with a DNA substrate carrying a synthetic analog of an AP site (2-hydroxymethyl-3-hydroxytetrahydrofuran, also known as the F-site [9]) and (ii) interaction with DNA that is a product of an exonuclease reaction and has an intact nucleotide (cytidine) in the enzyme’s active site [19], then it is obvious that both Nfo and DNA undergo similar conformational rearrangements (Figure 1). It has been shown that DNA is bent in the enzyme–substrate complexes of both types, while the damaged or 3′-terminal nucleotide is everted from the double helix and is situated in the active site. In this context, the opposite nucleotide located in the complementary strand is also flipped out of the DNA duplex. It must be noted that the 5′-phosphate group being hydrolyzed remains in the same position during either the AP endonuclease or the 3′-5′-exonuclease activity of Nfo, thereby ensuring the hydrolysis of the phosphodiester linkage. In addition, if the cytosine located in the enzyme’s active site is replaced with another nitrogenous base, then there are no steric hindrances, suggesting that the 3′-5′-exonuclease activity of Nfo is nonspecific to all the natural nitrogenous bases.

Recently, the mechanism behind the recognition of a target nucleotide by Nfo was examined by pre-steady-state kinetic analysis, pulsed electron–electron double resonance spectroscopy, and Forster resonance energy-transfer-based detection of conformational changes in DNA during DNA binding [20]. A real-time analysis of conformational alterations of DNA during its interaction with Nfo uncovered an increase in distances between duplex termini during the formation of the initial substrate–enzyme complex. A comparison of the efficiency of endonucleolytic cleavage of model DNA substrates containing different target nucleotides allows for use to conclude that double-helix bending and unwinding by the target nucleotide is one of the major reasons for its indiscriminate recognition by the Nfo of a target nucleotide. Moreover, it was found [21] that kinetic parameters of the DNA-binding steps, giving rise to a catalytically competent substrate–enzyme complex, are also significantly dependent on the nature of the nucleotide located opposite a lesion.

In the present work, we analyzed and described the kinetic features of the 3′-5′-exonucleolytic activity of Nfo. The efficiency of hydrolysis of phosphodiester bonds in several model DNA substrates was determined by the gel electrophoresis separation of the substrate and reaction products. Conformational changes in DNA during 3′-5′-exonucleolytic digestion of the model DNA substrates were recorded in real-time as changes in the intensity of 2-aminopurine (aPu) fluorescence using the stopped-flow method. Next, mathematical processing of the experimental data was performed, kinetic schemes describing the dynamics of the conformational changes were determined, and rate constants were calculated for elementary steps corresponding to these schemes. The obtained data revealed that the rate-limiting stage of the enzymatic process is the dissociation of the reaction product from the enzyme active site. It is interesting to note that the stability of the terminal pair of nucleotides in the substrate did not affect the enzymatic reaction rate. It was found that 2′-deoxynucleoside monophosphates can effectively inhibit the 3′-5′-exonuclease activity of Nfo.

## 2. Results and Discussion

### 2.1. Model DNA Substrates

**Figure 2:**
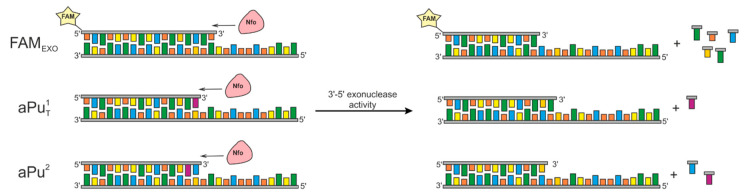
The model DNA substrates for studying the 3′-5′-exonuclease activity of Nfo.

The DNA duplexes formed by 15- or 17-mer oligodeoxyribonucleotides as a “short” strand and by complementary 28-mer oligodeoxyribonucleotides as a “long” strand were employed as model substrates in this study (Figure 2). According to the literature data, the structure with a 5′ single-stranded region is often used to examine this type of activity in AP endonucleases [22,23,24]. During the interaction between the enzyme and the DNA substrate, the 3′-terminal nucleotide is removed; then, the nucleoside monophosphate dissociates from the active site, and the enzyme moves in the 3′→5′ direction along the short strand to form a catalytic complex with the next nucleotide (meaning the sequential cleavage of 3′-terminal nucleotides).

The dye (FAM) that is chemically attached to the 5′ terminus of the degradable strand served as a fluorescent label to visualize the initial substrate and its degradation products in a PAAG. To register the removal of 3′-terminal nucleotides using the stopped-flow method, we used DNA substrates containing an aPu residue as a fluorophore located at either the first or second position from the 3′ end of the “short” strand (see substrates aPu^1^ and aPu^2^, respectively). It is known [25,26,27] that, within a DNA duplex, the fluorescence intensity of aPu is quenched after the formation of contacts (stacking or W-C pairing) with neighboring bases. Therefore, it is likely that the fluorescence intensity aPu will increase during the assembly of a substrate–enzyme complex, the cleavage of a nucleotide containing aPu, and its subsequent dissociation from the active site. In this context, the removal of aPu from the first position will occur immediately upon the formation of the catalytic complex, while the removal of aPu from the second position should be accompanied by the removal of the previous nucleotide and its dissociation from the active site.

### 2.2. Accumulation of 3′-5′-Exonucleolytic-Degradation Products

**Figure 3:**
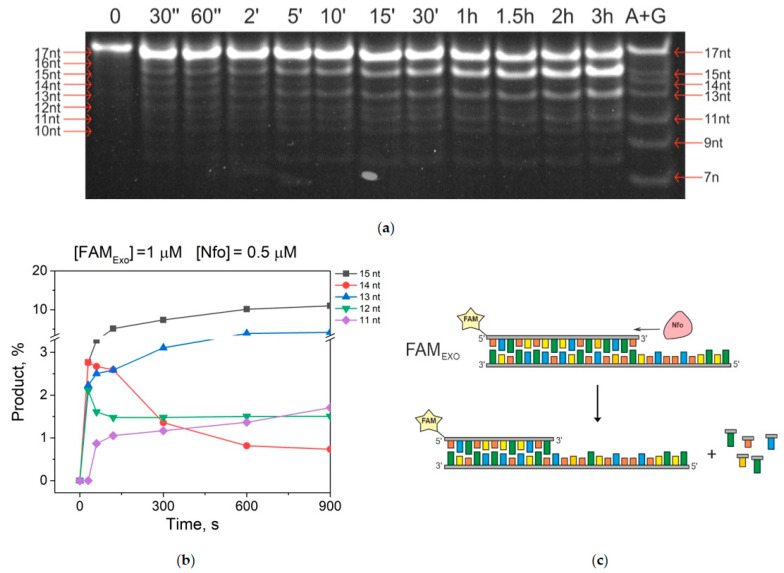
Visualization of the original DNA substrate (lane 1) and its degradation products after interaction with Nfo (lanes 2–12). Cleavage of substrate DNA at purine bases using the Maxam–Gilbert method (lane 13) (**a**). Kinetics of the accumulation and consumption of intermediate products of sequential cleavage of 3′-terminal nucleotides (**b**). Schematic representation of the interaction of Nfo with a DNA substrate accompanied by sequential cleavage of 2′-deoxynucleoside monophosphates from the 3′ end of the short strand. [FAM_Exo_] = 1.0 μM and [Nfo] = 0.5 μM (**c**).

To assess the rate of formation and distribution of products, curves in the accumulation of products during the interaction of Nfo with FAM_Exo_ were built with the help of gel electrophoresis (Figure 3a). To determine the length of the exonucleolytic-degradation products, the control lane of substrate DNA A+G cleavage at purine nucleotides was obtained using the Maxam–Gilbert method (lane 13).

The data showed that the removal of the first 3′-terminal nucleotide and the formation of a 16-mer product occurred during the first 30 s of the reaction and we were unable to record its accumulation (Figure 3a). After the initial 30 s of reaction, the 16-mer product did not accumulate; in contrast, the accumulation of the 15-mer product was detected for up to 3 h. The 16-mer product is not completely consumed, even within 3 h, and further degradation of the 15-mer product also proceeds very slowly. It is worth noting that the 14-mer product is only detectable in the reaction mixture during the first 5 min, after which it is completely converted into the 13-nt product, which also accumulates.

The loss of enzyme processivity after the cleavage of the first 3′-terminal nucleotide suggests that the enzyme is considerably inhibited by the reaction product. It could be hypothesized that the cleaved 3′-terminal nucleoside monophosphate blocks the active enzyme site and prevents the emergence of a new catalytic complex with the truncated product. Moreover, the differences in the consumption kinetics of the 14-mer product compared to the 15-mer and 16-mer products implies that each nucleoside monophosphate, when situated within the active site of this enzyme, has a distinct inhibitory effect. Thus, the 3′-5′-exonuclease activity of Nfo is context-specific and depends on the nature of the cleaved nucleotide.

### 2.3. Interaction with the DNA Substrate Containing aPu at the First Position

**Figure 4:**
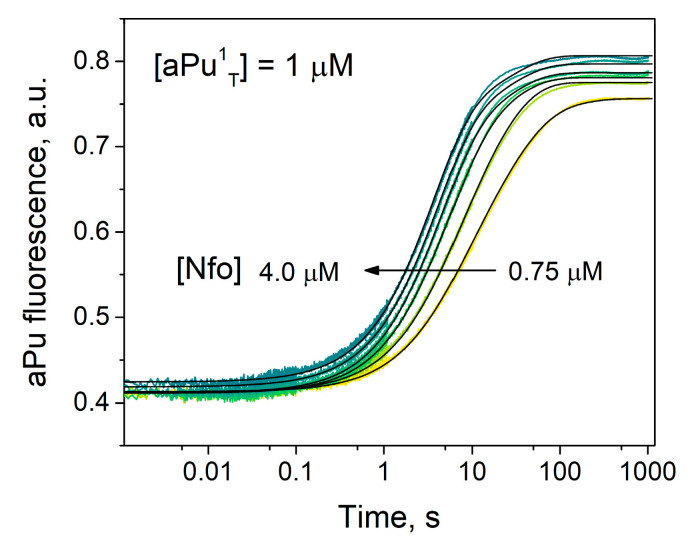
Alterations in the fluorescence intensity of aPu during the interaction of Nfo with aPu^1^_T_. [aPu^1^_T_] = 1.0 μM; Nfo concentration varied from 0.75 to 4.0 µM.

Regarding the interaction with the substrate called aPu^1^_T_ (containing aPu at the first position), the growth in fluorescence intensity was recorded in the time range from time point 100 ms to approximately second 10 or second 100, depending on the concentration of the enzyme (Figure 4). The obtained fluorescent kinetic curves are in good agreement with the data of gel electrophoresis and confirm that the removal of the 3′-terminal first nucleotide occurs within the initial 30 s of the reaction.

**Scheme 1:**
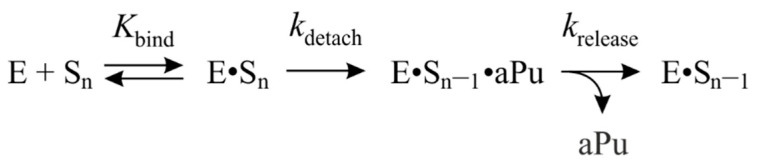
The kinetic mechanism of the interaction of Nfo with substrate aPu^1^_T._

When a kinetic scheme describing the cleavage of the first nucleotide was selected using the nonlinear regression method, a mechanism was proposed, which includes a reversible stage of enzyme binding to the substrate and two subsequent irreversible stages, which are probably the cleavage and release of aPu. This kinetic mechanism (Scheme 1) contains the lowest possible number of stages to describe the experimental kinetic curves. The rate constants characterizing the stages of this kinetic scheme are presented in Table 1. It is worth noting that the rate constant of aPu cleavage is more than 10 times higher than the rate constant of its release from the active site of this enzyme.

### 2.4. The Influence of Stability of a 3′-Terminal Base Pair on Binding and Cleavage Efficiency

**Figure 5:**
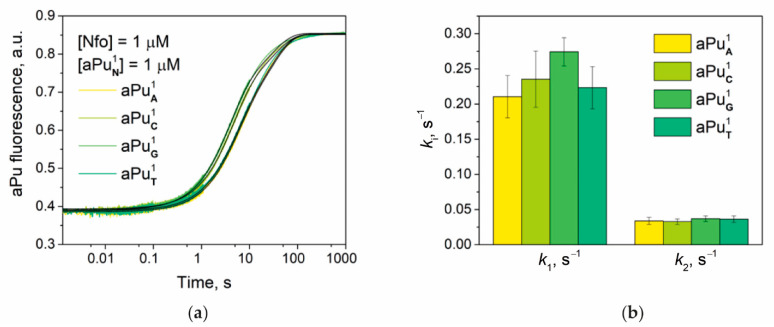
Changes in the fluorescence intensity of aPu as a consequence of the interaction between Nfo and aPu^1^_N_ (**a**). Observed rate constants *k_obs_*^1^ (**b**). The data were fitted to the exponential function described by Equation (1) (**a**).

Another possible factor that can affect the formation efficiency of the catalytic enzyme–substrate complex and the rate of removal of various nucleotides is the stability of a 3′-terminal base pair. To test this suggestion, a set of DNA substrates was tested that contained aPu opposite to all the natural nucleotides. It is known that the thermal stability of DNA duplexes containing aPu decreases in the series aPu/T > aPu/C > aPu/A > aPu/G [28]. The obtained kinetic curves and the corresponding observed rate constants (Figure 5a,b) indicate that the stability of the terminal pair of nucleotides has almost no impact on the removal rate of the terminal nucleotide. Therefore, this finding supports the hypothesis that the dissociation of nucleoside monophosphate out of the active site is rate-limiting step.

### 2.5. Interaction with the DNA Substrate Containing aPu at the Second Position

**Figure 6:**
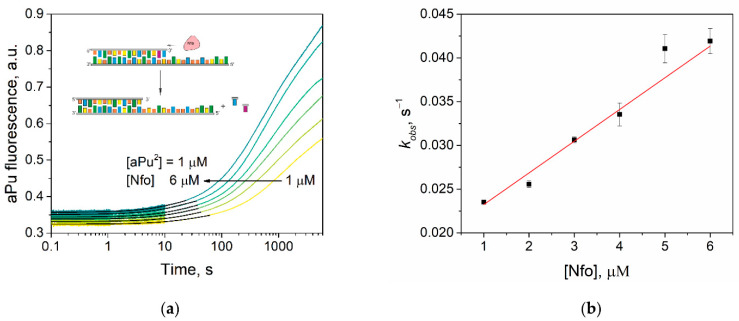
Changes in the fluorescence intensity of aPu during the interaction between Nfo and aPu^2^ (**a**). The Nfo concentration dependence of the observed rate constant (**b**). [aPu^2^_T_] = 1.0 µM; Nfo concentration varied between 1.0 and 6.0 M.

As in the previous case, the interactions between Nfo and this substrate (Figure 6a) are accompanied by the growth in fluorescence intensity, but the latter process is much slower and does not end within 1 h, even in the presence of a sixfold excess of the enzyme.

**Scheme 2:**
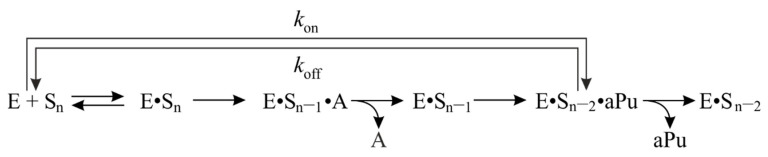
The kinetic mechanism of the interaction between Nfo and substrate aPu^2.^

To estimate the rate constant for the cleavage of the second nucleotide, the initial portion of these kinetic curves was fitted to an exponential function according to Equation (1). The observed rate constants manifested a linear dependence on the enzyme concentration (Figure 6b). This type of dependence indicates that the process characterized by these constants is bimolecular and reversible (Table 2). The constants obtained in accordance with Equation (2) characterize a complex multistep process containing the binding of this enzyme to this substrate, the cleavage and release of the first 3′-terminal nucleotide, and the cleavage of the second (next) nucleotide (Scheme 2).

### 2.6. Determination of the Inhibitory Ability of 2′-Deoxynucleoside Monophosphates

**Figure 7:**
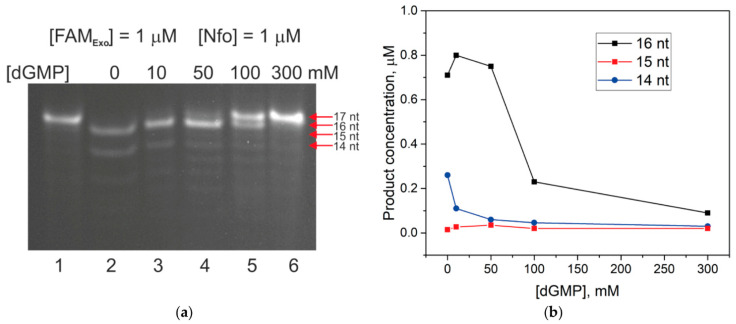
The ratio of the initial DNA substrate to products during the interaction between Nfo and FAM_Exo_ in the presence of 2′-deoxyguanosine monophosphate (**a**). Amounts of 16- and 15-mer products in the reaction mixture as a function of dGMP concentration (**b**). [Nfo] = 1.0 μM and [FAM_Exo_] = 1.0 μM; [dGMP] varied from 0 to 300 mM.

Our set of findings indicates that the rate-limiting step of the enzymatic process is the release of nucleoside monophosphate from the enzyme active site. Accordingly, the products of the Nfo interaction with the DNA substrate were visualized by gel electrophoresis in the presence of different concentrations of 2′-deoxyguanosine monophosphate (from 10 to 300 mM; Figure 7a). It is obvious that, after the nucleoside concentration was raised, there was a pronounced slowdown of the exonucleolytic degradation of the substrate (Figure 7b).

**Figure 8:**
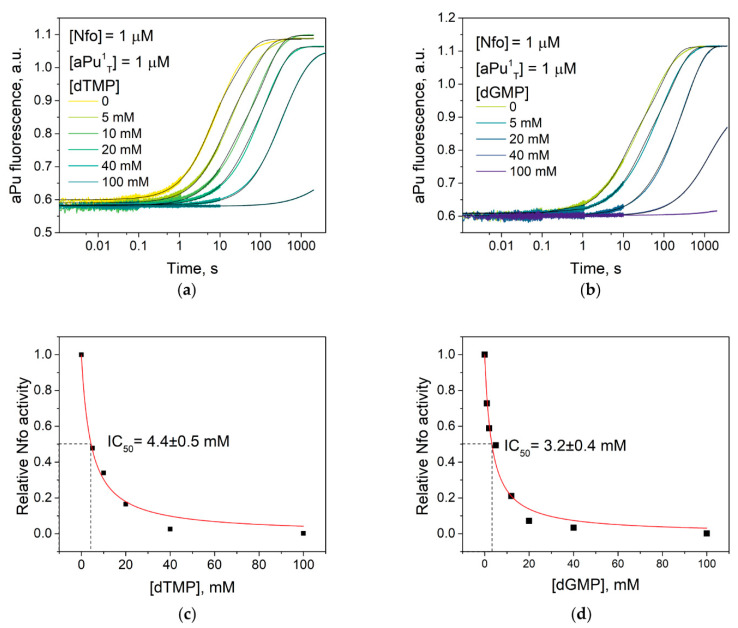
Changes in aPu fluorescence intensity over the course of interactions between Nfo and aPu^1^_T_ in the presence of dGMP (**a**) or dTMP (**b**). [aPu^1^_T_] = 1.0 μM and [Nfo] = 1.0 μM. The decline in Nfo activity depending on the concentration of dGMP (**c**) or dTMP (**d**) in the reaction mixture.

To quantitatively describe this process, curves were constructed for changes in aPu fluorescence intensity using the stopped-flow method; these curves characterize the interaction between Nfo and aPu^1^_T_ in the presence of either 2′-deoxyguanidine monophosphate or thymidine monophosphate at various concentrations (Figure 8a,b). It was demonstrated that, at a higher concentration of 2′-deoxynucleoside monophosphates, the growth in aPu fluorescence intensity slows down, indicating the inhibition of the enzymatic reaction. The curves were fitted to the exponential function described by Equation (1). The resultant rate constants showed a hyperbolic dependence on the concentration of 2′-deoxynucleoside monophosphates (Figure 8c,d). From this dependence, IC_50_ values (inhibitor concentration at which the enzymatic activity diminished by 50%) were calculated for 2′-deoxyguanidine monophosphate and thymidine monophosphate: 3.2 ± 0.4 and 4.4 ± 0.5 mM, respectively.

## 3. Conclusions

In this work, the pre-steady-state kinetics of the interaction between *E. coli* AP endonuclease Nfo and various intact DNA duplexes with a 5′ single-stranded region was investigated for the first time. It was demonstrated that the cleavage rate of the 3′-terminal nucleotide is significantly affected by its nature, suggesting that the 3′-5′-exonuclease activity of Nfo is context-specific. In contrast, the stability of the terminal base pair has virtually no impact on the cleavage rate of the 3′-terminal nucleotide. Kinetic schemes are proposed, which characterize the cleavage of the first and second nucleotide from the 3′ end of the “short strand,” along with the rate constants of the corresponding steps. It was established that a rate-limiting step in the enzymatic process is the dissociation of the cleaved 2′-deoxynucleoside monophosphate from the active site of the enzyme. In this context, 2′-deoxynucleoside monophosphates can effectively inhibit the 3′-5′-exonuclease activity of Nfo.

## 4. Materials and Methods

In this study, various domestically manufactured reagents of a high purity grade and chemicals from Sigma-Aldrich were used: acrylamide, N,N′-methylenebisacrylamide, urea, glycerol, tris(hydroxymethyl)aminomethane, EDTA, dithiothreitol, KCl, ZnCl_2_, HCl, NaOH, ammonium persulfate, tetramethylethylenediamine, agar, tryptone, yeast extract, isopropyl-β-D-1-thiogalactopyranoside (IPTG), and bovine serum albumin (BSA). All solutions were prepared using twice-distilled water.

### 4.1. The Enzyme and Oligonucleotides

Oligonucleotides modified with 2-aminopurine (aPu) or 6-carboxyfluorescein (FAM) within DNA duplexes were used as DNA substrates (Table 3).

The Nfo enzyme was isolated from cells of *E. coli* strain BL21(DE3) (Invitrogen, France), transformed with the pET-11a plasmid containing the *nfo* gene, as described previously [18]. Experiments were conducted at 37 °C in a buffer consisting of 50 mM Tris-HCl (pH 7.5), 100 mM KCl, 1 mM EDTA, 1 mM dithiothreitol, and 7% of glycerol.

### 4.2. Polyacrylamide Gel (PAAG) Electrophoretic Analysis of the Cleavage of DNA Substrates

To build curves for the accumulation of reaction products, equal volumes of 2.0 μM FAM_Exo_ substrate and a 1.0 μM Nfo solution were rapidly mixed at 37 °C. Aliquots of 10 µL were taken from the reaction mixture at different timepoints. The enzymatic reaction was stopped by the addition of an equal volume of formamide containing 25 mM EDTA and 0.1% of xylene cyanol, followed by incubation at 95 °C for 3 min. After that, the reaction mixture was loaded to a denaturing 20% PAAG, and electrophoresis was carried out at a voltage of 50 V/cm. The resultant gel was visualized in an E-Box-CX5 gel documentation system. The degree of substrate cleavage was determined as the ratio of peak areas of a product to the sum of peak areas of the product and the peak area of the initial oligonucleotide in the Gel-Pro Analyzer software. All kinetic curves shown in the figures are the average of three traces obtained in individual experiments.

To assess the inhibitory ability of 2′-deoxyguanosine monophosphate by gel electrophoresis, 2′-deoxyguanosine monophosphate was added to Nfo and FAM_Exo_ solutions beforehand, at a concentration of from 0 to 300 mM. Then, the solutions were incubated for 30 min at 4 °C, after which the solutions of the enzyme and the substrate DNA were mixed, and the reaction mixture was kept for 30 min at 37 °C. Concentrations of the enzyme and DNA substrate in the reaction mixture were 1.0 μM. Next, the reaction products were analyzed by PAAG electrophoresis.

### 4.3. Kinetic Analysis of the Cleavage of DNA Substrates by the Stopped-Flow Method

The registration of fluorescent kinetic curves was conducted by the stopped-flow method on an SX.18MV instrument (Applied Photophysics, Leatherhead, UK). The method is based on a rapid mixing of the reactants, followed by flow stoppage, with continuous recordings of the fluorescence intensity. The SX.18MV instrument is equipped with a xenon lamp as an excitation source. The dead time of the instrument was 1.3 ms. aPu was employed as a fluorophore. The data acquisition was performed under conditions close to one turnover of the enzyme, i.e., the enzyme concentration and substrate concentration were similar (within an order of magnitude).

To obtain the concentration series of kinetic curves of changes in fluorescence intensity of aPu, the solution containing Nfo was quickly mixed in the reaction chamber with a solution of DNA substrate aPu^1^_T_ or aPu^2^. The concentration of Nfo in the reaction mixture varied from 0.75 to 6.0 μM. Each kinetic curve was averaged from the results of at least three experiments. To assess the effect of the stability of the 3′-terminal base pair on the rate of 3′-5′-exonucleolytic DNA degradation, a DNA duplex aPu^1^_N_ was used: a substrate containing each of the four types of natural nucleotides opposite aPu. To evaluate the inhibitory ability of 2′-deoxynucleoside monophosphate, we added to the Nfo and aPu^1^_T_ solutions either 2′-deoxyguanosine monophosphate or 2′-deoxythymidine monophosphate, at concentrations 0 to 100 mM. Next, the solutions were incubated for 30 min at 4 °C, after which the reaction was carried out in the manner described above. The concentrations of Nfo and the DNA substrate in the reaction mixture were 1 μM unless stated otherwise.

In order to calculate the rate constants of conformational transitions, OriginLab 15.0 (OriginLab Corp., Northampton, MA, USA) and DynaFit 4 software (Biokin, Pullman, WA, USA) [29] were utilized to compute the parameters of the catalytic process (kinetic constants) via the nonlinear regression method. The latter involved the numerical integration of a system of differential equations describing the kinetic mechanism of the reaction, as explained earlier [30,31].

To calculate the observed rate constants characterizing the change in fluorescence intensity, the following equation was used in the OriginLab 15.0 software:

F = F_0_+∑A_i_ × [1 − exp(−***k***_obs_^i^ × t)] (1)

where F is experimental fluorescence, F_0_ denotes background fluorescence, A_i_ represents fluorescence change amplitude, and ***k***_obs_^i^ is the observed rate constant. The R-Squared value in all fits was no lower than 95.

The dependence of observed rate constants ***k***_obs_ on the initial Nfo concentration was fitted to the following linear equation:

***k***_obs_ = ***k***_on_ × [Nfo] + ***k***_off_ (2)

## Figures and Tables

**Table 1:** Rate constants for the interaction of Nfo with aPu^1^_T._

Rate Constant	aPu^1^_T_
*K*bind, 10^−6^ M^−1^	0.67 ± 0.02
*k*detach, s^−1^	0.54 ± 0.01
*k*release, s^−1^	0.039 ± 0.001

**Table 2:** Rate constants for interaction between Nfo and aPu^2^.

Rate Constant	aPu^2^
*k*on, M^−1^ s^−1^	3600 ± 400
*k*off, s^−1^	0.020 ± 0.001
*K*, 10^−6^ M^−1^	0.18 ± 0.02

**Table 3:** The model DNA substrates used in this work.

Name	Sequence	Structure
aPu^1^_N_ N = A, C, G, T	5′-CAGCTCTGTACGTG(**aPu**)-3′ 3′-GTCGAGACATGCAC **N** CGTCACCACTGTG-5′	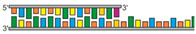
aPu^2^	5′-CAGCTCTGTACG(**aPu**)A-3′ 3′-GTCGAGACATGCA C **T**CGTCACCACTGTG-5′	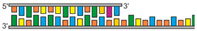
FAM_Exo_	5′-FAM-CAGCTCTGTACGTGAGC-3′ 3′-GTCGAGACATGCACTCGTCACCACTGTG-5′	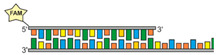

## Data Availability

Raw experimental data are available from N.A.K. upon request. Tel.: +7-(383)-363-5174, E-mail: nikita.kuznetsov@niboch.nsc.ru.
